# Expression levels of plasma exosomal miR-124, miR-125b, miR-133b, miR-130a and miR-125b-1-3p in severe asthma patients and normal individuals with emphasis on inflammatory factors

**DOI:** 10.1186/s13223-021-00556-z

**Published:** 2021-05-17

**Authors:** Mostafa Atashbasteh, Esmaeil Mortaz, Seyed Alireza Mahdaviani, Hamidreza Jamaati, Abdolamir Allameh

**Affiliations:** 1 Department of Clinical Biochemistry, Faculty of Medical Sciences, Tarbiat Modares University, Tehran, Iran; 2 Department of Immunology, Faculty of Medicine, Shahid Beheshti University of Medical Sciences, Tehran, Iran; 3 Clinical Tuberculosis and Epidemiology Research Center, National Research Institute of Tuberculosis and Lung Diseases, Shahid Beheshti University of Medical Sciences, Tehran, Iran; 4 Pediatric Respiratory Diseases Research Center, National Research Institute of Tuberculosis and Lung Diseases (NRITLD), Shahid Beheshti University of Medical Sciences, Tehran, Iran; 5 Chronic Respiratory Diseases Research Center, National Research Institute of Tuberculosis and Lung Diseases, Shahid Beheshti University of Medical Sciences, Tehran, Iran

**Keywords:** Severe asthma, Exosomal fraction, Micro RNA, CRP, IgE

## Abstract

**Background:**

Identification of molecular markers, such as miRNAs is promising for the diagnosis of asthma and its clinical phenotypes. The aim of this study was to examine the changes in the expression of selected microRNAs in plasma exosomal fractions of severe asthma patients. The expression of miRNAs was determined in relation to the changes in inflammatory markers.

**Method:**

Severe asthma patients (n = 30) and healthy subjects (n = 30) were selected among the individuals referred to asthma and allergy clinic. Blood was collected from each participant to determine the serum high-sensitive C-reactive protein (hs-CRP) and total IgE. The exosomal fraction of plasma was isolated and processed for quantitation of miR-124, miR-125b, miR-133b, miR-130a and miR-125b-1-3p expression using quantitative real time-PCR (qRT-PCR).

**Results:**

Serum hs-CRP and total IgE were significantly higher in asthma patients compared to controls. Expression of miR-124, miR-133b, and miR-130a was down-regulated in asthma patients as compared to controls (p < 0.0001). However, the expression of miR-125b was substantially higher in patients compared to controls (p < 0.0001). There was no significant difference in the expression of miR-125b-1-3p in the patients and controls. Data analysis revealed that among the miRNAs, changes in miR-125b in severe asthma patients were highly correlated with the serum levels of hs-CRP and IgE.

**Conclusion:**

Overexpression of miR-125b in severe asthma which was associated with serum IgE and hs-CRP may suggest that this molecule is linked to inflammatory reactions. Up-regulation of miR-125b together with decreased expression of miR-124, miR-133b, and miR-130a may suggest that this miRNA profile is useful for diagnosis and discrimination of clinical phenotypes of asthma.

## Introduction

Asthma is a common inflammatory disease distinguished by intermittent airflow obstruction, remodeling, and airway inflammation, affecting 1–18% of the population in different countries. It is characterized by variable and recurring symptoms of wheezing, coughing, chest tightness, and variable expiratory airflow limitation [1]. The prevalence of asthma has been significantly increased in the twentieth century [2] and three clinical phenotypes have been defined as; mild persistent, moderate persistent, and severe persistent asthma [3].

According to the GINA classification, the majority of the asthma patients such as mild asthma (step 1 or step 2), moderate asthma (step 3) and severe asthma (step 4 or 5) respond to anti-inflammatory treatments [1].

While most GINA step 4 and 5 can be adequately controlled (often at the expense of very high doses of ICS/OCS), a significant proportion of these patients are sub-optimally controlled.

Asthma may occur due to an inflammatory response by activation of various inflammatory cells [4–7]. In fact, the correlation between inflammatory cells that produce biomolecules and airway smooth muscle cells (ASM) is a causal factor in the pathophysiology of asthma [8]. Hence, the molecules and intermediates that couple with signal transduction pathways play important role in the pathogenesis of asthma. The presence of different clinical asthma phenotypes provides this opportunity to discuss various molecular functions especially cytokines, including interleukins, and other biomolecules in asthma pathogenesis. Immunoglobulin E (IgE) and highly-sensitive C-reactive protein (hs-CRP) are two mediators that are associated with inflammatory diseases such as asthma [9–11]. The binding of allergens to mast cells or basophilic cells may lead to cross-linking of the IgE on the cell surface, leading to the release of factors which mediates the reaction so-called type-1 hypersensitivity. In patients suffering from asthma, the potential of serum hs-CRP has been shown in recognizing the inflammatory process and increased serum hs-CRP may be used for assessment of treatment in inflammatory conditions [12].

Increased levels of sphingosine 1-phosphate (S1P) in the airways of asthmatic patients exacerbate the ASM hyper-responsiveness during the inflammation of the airways in a mast cell-dependent manner [13]. Therefore bioactive molecules such as S1P as a lipid mediator which acts as a messenger in various intercellular communications can play important role in airway inflammation and asthma [14]. S1P is generated from sphingosine through the actions of the sphingosine kinase1 and 2 (SphK1/2) isoenzymes.

Exosomes are 30–120 nm membrane-enclosed extracellular vesicles, released from most cell types into the extracellular space which can be detected in almost all human body fluids [15–20]. Exosomes shuttle biological molecules including protein, lipid, nucleic acids and microRNAs between cells. These extracellular vesicles play an important role in cell to cell communication [21]. It has been reported that under pathological conditions such as inflammation, dyspnea, airflow obstruction, and hypoxic pulmonary hypertension the content of exosomal microRNAs (miRNAs) may alter which might affect the cell stress response[22, 23].

The miRNA molecules are a group of small (18–22 nucleotides), single-stranded, non-coding RNAs play a critical role in the regulation of gene expression and immune system functions. miRNAs also have been reported to affect both the stability and the translation of mRNAs by base-pairing with the complementary sequences within mRNA molecules and effects on methylation or targeting of transcription factors [24–26].

A group of miRNAs has been shown that target Sphingosine-1-phosphate (S1P) signaling pathway. Among these, miR-124 targets the Sphingosine Kinase 1 (SphK1) [27], miR-133b and miR-125b-1-3p bind to the S1P receptor 1 (S1PR1) [28, 29], miR-130a-3p targets S1P receptor 2 (S1PR2) [30], and miR-125b targets S1P lyase 1 (SGPL1) messenger RNAs [31].

Emerging of reports show the existence of exosomes in bronchoalveolar lavage fluid (BALF) of asthmatics patients containing miRNAs that could be responsible for the induction of inflammatory responses leading to bronchial hyper-responsiveness [22, 32].

The conventional markers used for early diagnosis of asthma including, pulmonary function tests are often insensitive and the use of bronchial biopsies for investigation of airway inflammation are invasive and costly to perform in clinics. Several other markers such as pH, nitric oxides, cytokines, leukotrienes, isoprostanes, and other biomolecules are considered as non-invasive and valuable techniques in the diagnosis and management of asthma [33, 34]. The development of molecular markers, particularly circulating miRNAs is an emerging issue that could be useful for the diagnosis of asthma and discrimination of asthma clinical phenotypes. Hence, changes in the expression of exosomal miRNA in the blood could be promising for differential diagnosis of asthma and other inflammatory diseases. In addition, the use of miRNAs as biomarkers could be considered as a novel approach for diagnosis and monitoring the treatment of asthma [35, 36].

The specificity of miRNAs to asthma and its clinical phenotypes is possible by understanding the expression of each miRNA with its relevant pathway or the inflammatory reactions which are linked to the progression of the disease. In this line, Panganiban and co-workers have identified 30 miRNAs that were differentially expressed among healthy, allergic, and asthmatic subjects. These miRNAs have been grouped into 5 different expression patterns. In the case of asthmatic patients, 2 subtypes have been identified that differed by high or low peripheral eosinophil levels. This finding showed that the circulating miR-125b, miR-16, miR-299-5p, miR-126, miR-206, and miR-133b levels were most predictive of allergic and asthmatic status [37].

The aim of the present study was to identify miRNAs that may differentially be expressed in severe asthma patients. Therefore, based on the microRNA databases some miRNAs which are believed to be the target genes and pathways in inflammatory reactions have been selected. Then the expression levels of miR-124, miR-125b, miR-133b, miR130a, and miR-125b-1-3p, were compared in exosomes isolated from plasma samples of a group of severe asthma patients and a normal control group. These changes were analyzed in relation to serum IgE and high sensitive C-reactive protein (hsCRP) levels.

## Methods

In this case–control study, 30 patients diagnosed as severe asthma (aged 23–60 years) were selected among the individuals referred to the Asthma and Allergy Clinic (Masih Daneshvari Hospital, Tehran, Iran). All the patients were examined and diagnosed with severe asthma by the clinicians according to the clinical guidelines of the Global Initiative for Asthma (GINA, 2019). Non-asthmatic-medical inflammatory disease, and subjects suffering from infections like pneumonia, smokers, and those with any type of systemic disease, and neoplasms were excluded from the cases.

In addition, 30 individuals with an age range of 25–58 years (matched to patients) were selected and considered as the normal control group. These individuals were also referred to the same clinic from the industrial sector for a periodic checkup. The individuals in the normal control group showed no signs or symptoms of allergy or asthma. As shown in Table 1, normal individuals had a normal complete blood count (CBC) and normal pulmonary function test (PFT). The exclusion criteria for the control group was the use of medications or corticosteroid drugs, individuals suffering from immune-mediated inflammatory disease (IMID), chronic obstructive pulmonary disease (COPD), infections such as pneumonia, lung cancer, other breathing problems, smokers, any type of systemic disease, and neoplasms.

**Table 1:** Demographic and clinical characteristics of the asthma group and normal control groups

	Control (n = 30)	Severe asthma (n = 30)	P-value
Age	39.23 ± 10.17	42.13 ± 9.41	*0.26*
Sex (M/F)	13/17(43.3/56.7%)	12/18 (40/60%)	–
Age of asthma onset (Year)	–	31.90 ± 10.55	
FEV1 (% predicted) ± SD	99.19 ± 10.99	51.37 ± 16.10	< *0.0001*
FVC (% predicted) ± SD	94.02 ± 8.39	63.50 ± 14.84	< *0.0001*
FEV1/FVC	0.85 ± 0.59	0.69 ± 0.11	< *0.0001*
BMI (Mean ± SD)	22.46 ± 2.28	23.30 ± 2.04	*0.14*
Blood eosinophil X 10^9^ per L	0.09 ± 0.04	0.52 ± 0.21	< *0.0001*
Medication requirements (n) ICS(LABA and LAMA) / ICS + OCS	–	10/20	–
Atopic (%)	Non	80.0 (n = 24)	–
Serum IgE IU/mL	39.32 ± 5.33	232.8 ± 25.79	< *0.0001*

Asthma patients were divided into two groups according to the type of medication. One group was under Inhaled corticosteroid (ICS) and the other group was under ICS + Oral corticosteroid (OCS) therapy

Normal controls had no history of predisposing to any chronic or inflammatory disease. Forced expiratory volume in 1 s (FEV1%) and Forced vital capacity (FVC %) detected. Atopic Determined by allergen skin prick tests. The p-value < 0.05 is considered significant

This study has been approved by the Medical Ethics Committee of the Tarbiat Modares University (ID: IR.MODARES. REC.1397.047). Blood samples were collected from all the patients and normal controls from May 2018 to July 2019. Approximately 5 mL of peripheral intravenous blood specimen was collected from each individual after obtaining a signed informed consent. The sample was divided into two aliquots, one in a tube containing an anticoagulant (K3-EDTA), and another aliquot was collected in a clot tube (with gel separator).The sample collected in a K3-EDTA tube(3 mL) was used for exosome separation. For this, plasma was separated by centrifugation at 1600×*g* for 10 min at room temperature. Plasma was filtered through a 0.22-μm syringe filter before transferring into a new tube. Plasma was further centrifuged at 16,000×*g* for 10 min at room temperature to remove residual dead cells, cellular debris, and apoptotic bodies as described earlier [39]. The blood sample collected in a clot tube (2 mL) was centrifuged at 302×*g* for 15 min at 4 °C and then serum was separated and used for estimation of hs-CRP and total IgE as described.

### Estimation of serum CRP and IgE

The total serum IgE was measured in all the samples by electrochemiluminescence immunoassay (ECLIA) technique using fully automated immunoassay analyzer (Cobas e 411, Roche Diagnostics GmbH, Germany) following the manufacturer’s instruction. IgE concentration was considered positive at > 100 IU/mL for adults as recommended (Biomedical Laboratory Inc.)[40].

Serum hs-CRP- was also estimated based on turbidimetric assay on COBAS INTEGRA® 400 plus (Roche Diagnostic, Mannheim, Germany) autoanalyzer at a wavelength of 552 nm. The reference interval was < 5 mg/L for adults (Biomedical Laboratory Inc.) [41, 42].

### Isolation of exosomes from cell-free plasma

Exosomes were isolated from cell-free plasma samples by ultracentrifugation as described by Théry et al. [43] Briefly, 500 µl of plasma sample was diluted with an equal volume of phosphate-buffered saline (PBS) and centrifuged for 30 min at 12,000×*g* at 4 °C. The supernatant was separated and transferred into an ultracentrifuge tube and centrifuged for 70 min at 100,000×*g* at 4 °C (VS-35SMTi, Vision Scientific Co. Ltd., South Korea). The pellet obtained from each sample was re-suspended in 1 ml of PBS, and centrifuges again at 100,000×*g* at 4 °C for 70 min to wash the pellet. The supernatant was discarded and the exosomal fraction was collected and re-suspended in 150 μL PBS and stored at − 80 °C for further use.

### Protein determination

The total protein content of the exosomal fraction was measured using the BCA Protein Assay kit (Thermo Scientific Pierce™, USA). This method is based on bicinchoninic acid for the colorimetric detection of total protein according to the manufacturer's instruction. Finally, the protein concentration was calculated using a linear standard curve drawn with bovine serum albumin (BSA) standard provided with the assay kit.

### Characterization of the exosomes

Different approaches were used to check the quality and quantity of the isolated exosomes. The samples were subjected to the dynamic light scattering method (DLS) with Zetasizer Instrument (Malvern Zetasizer, Nano Series, The UK). Then the samples were subjected to Transmission electron microscopy (TEM) analysis. In this experiment, the exosome-containing solution was mixed with glutaraldehyde solution (2.5%) and dried onto 100 mesh carbon-coated TEM grids. After fixation, the grids were negatively stained with phosphotungstic acid (PTA) and imaged were recorded on a Zeiss-EM10C transmission electron microscopy (TEM, Zeiss–EM10C–100 kV, Germany) operating at an accelerator voltage of 100 kV.

The exosomes were also subjected to flow cytometry analysis as described earlier [44]. For this, 10 µg of exosomes was coupled with 20 μl of aldehyde/sulfate latex beads (4 μm in diameter from Invitrogen, The USA) for overnight at 4 °C on an orbital rotator for the blocking of remained sites. The product was then incubated with 100 mM glycine (Sigma-Aldrich, The USA) for 30 min and after washing with FACS buffer, stained with CD63-PE antibody or isotype control and CD81-FITC antibody or isotype control (eBioscience, San Diego, CA, The USA). Then 10,000 events were measured by a Flow cytometer (FACS Calibour, BD, USA). Data were analyzed using FlowJo software (FlowJo 7.6).

### RNA extraction and cDNA synthesis

Total RNA was extracted from each exosomal sample using TRIzol solution (Invitrogen, The USA). The exosomal RNA concentration (ng/µL) was quantified using the NanoDrop-2000 spectrophotometer (NanoDrop Technologies Inc., Wilmington, DE, The USA). Absorbance at 260 nm was used for the determination of total RNA concentration in the samples. The absorption ratio of 260/280 nm (1.8–2) and 260/230 nm (1.8–2.2) were used to assess RNA purity. The RNA samples were then resuspended in 20 μL DEPC water and stored at −20 °C for further use. Then 5 µg of each RNA sample was subjected to reverse transcription using the BON-miR miRNA 1st-Strand cDNA Synthesis Kit based on Poly (A)-Tailed universal Reverse Transcription method using poly(A) polymerase (PAP) as described earlier [45].

### Real-Time Quantitative PCR (qRT-PCR) for miRNA expression

In this experiment, 5 microRNA molecules were selected based on their target genes identified by the TargetScan, miRTarBase and miRDB databases, and based on the target genes.

The exosomal miRNA expression was determined using the BON-miR High-Specificity miRNA qPCR Kit and miRNA-specific primers (synthesized by Stem Cell Technology Research Center, Tehran, Iran, BON209001) on a LightCycler® 96 PCRsystem (Roche Applied Science, Germany). A two-step real-time PCRprotocol was adopted using an initial denaturation step of 2 min at 95 °C, followed by 40 amplification cycles including a denaturation step (5 s at 95 °C), and an annealing step (the 30 s at 60 °C) based on the kit brochure. The melting curve was prepared for all the reactions to confirm the precision of each sample. The cycle threshold (Ct) values for the samples were normalized to the U6 expression as an internal control and results were then converted into fold change using the 2^−ΔΔCt^ formula.

### Statistical analysis

The qPCR data were analyzed using The 2^-ΔΔCt formula by the Livak method[46]. All the assays were performed in duplicate and the results are presented as mean ± SD of samples from patients and normal individuals. The data were analyzed using the graph pad Prism Software (version 8.0). The differences between groups were compared by Student’s t-test and Mann Whitney U test for significance (p < 0.05). In other parts of the paper, data are shown as means (95% confidence intervals; 95% CI). The relationship between inflammatory factor concentrations and miRNAs expression in severe asthma patients was assessed by the Spearman’s rank correlation test.

## Results

### Demographic information

The demographic and clinical characteristics of the patient and normal control groups are summarized in Table 1. The PFT including Forced expiratory volume in 1 s (FEV1%) and Forced vital capacity (FVC %), Blood eosinophil count and serum IgE level were significantly higher in asthmatic patients as compared to that measured in normal individuals (p < 0.0001). In addition based on allergen skin prick tests which have been adopted for local environmental allergens approximately 80% of the patients showed atopic symptoms.

### Characterization of the isolated plasma exosomes

**Fig. 1:**
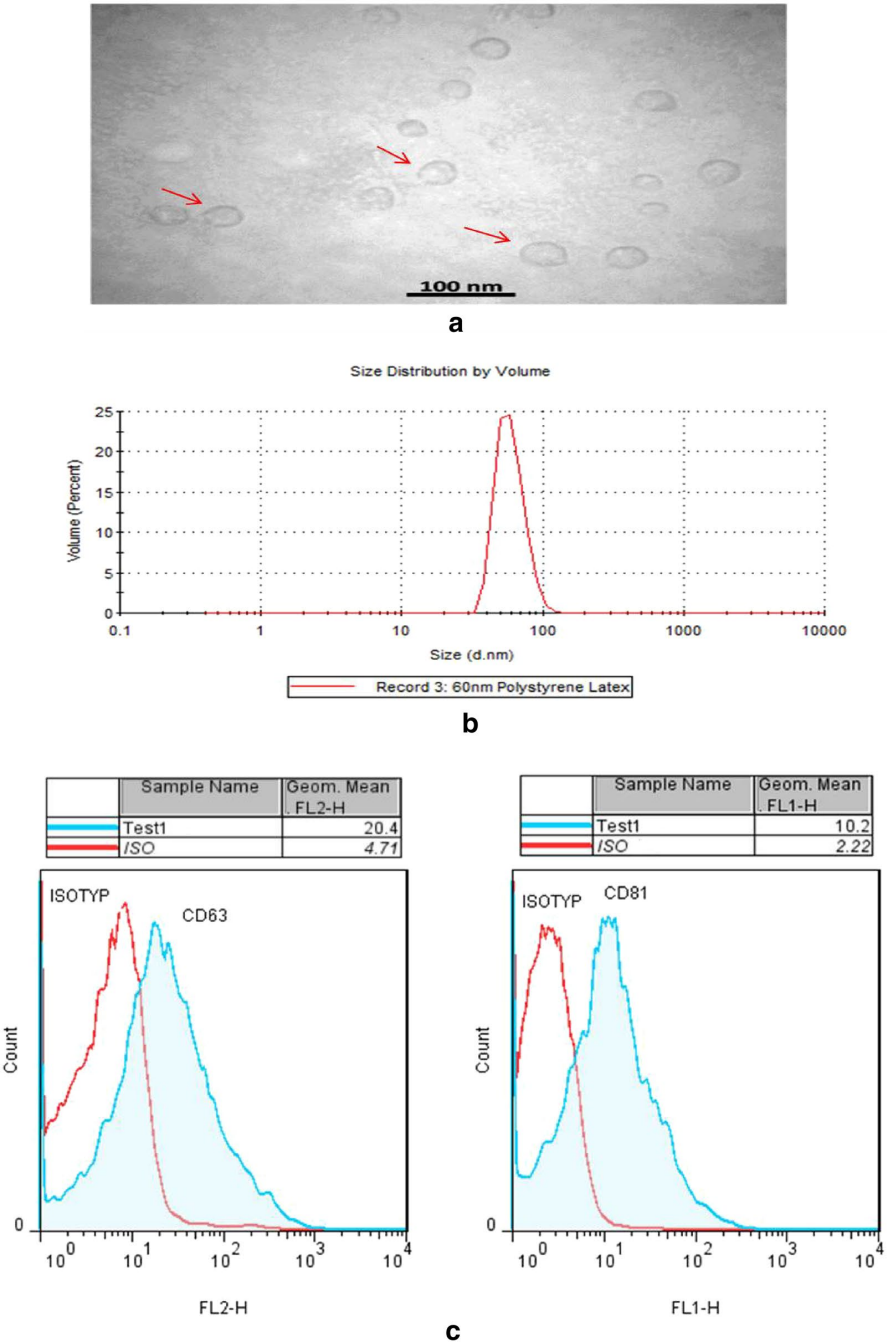
Characterization of the plasma-derived exosomes. Purification and characterization of plasma exosomes have been described in the ‘Methods’ section. **a** The arrows in the transmission electron microscopy (TEM) image show the spherical shape of the exosomes. Scale bar represents 100 nm. **b **Shows the size distribution of the exosomes analyzed by Dynamic light scattering. The average size (Z-Average) was 55 nm. **c** Flow cytometry data show the expression of surface markers on the exosomes; CD81 and CD63

The exosomes prepared from plasma samples of asthma patients and normal controls were found to be spherical shape particles as shown under TEM (Fig. 1a). The average size of the particles was 55 nm as determined by the DLS method (Fig. 1b). Flow cytometry analysis showed that the isolated exosomes were positive for CD81 and CD63 surface markers (Fig. 1c).

### Expression of the micro RNAs in the exosomal fractions

**Fig. 2:**
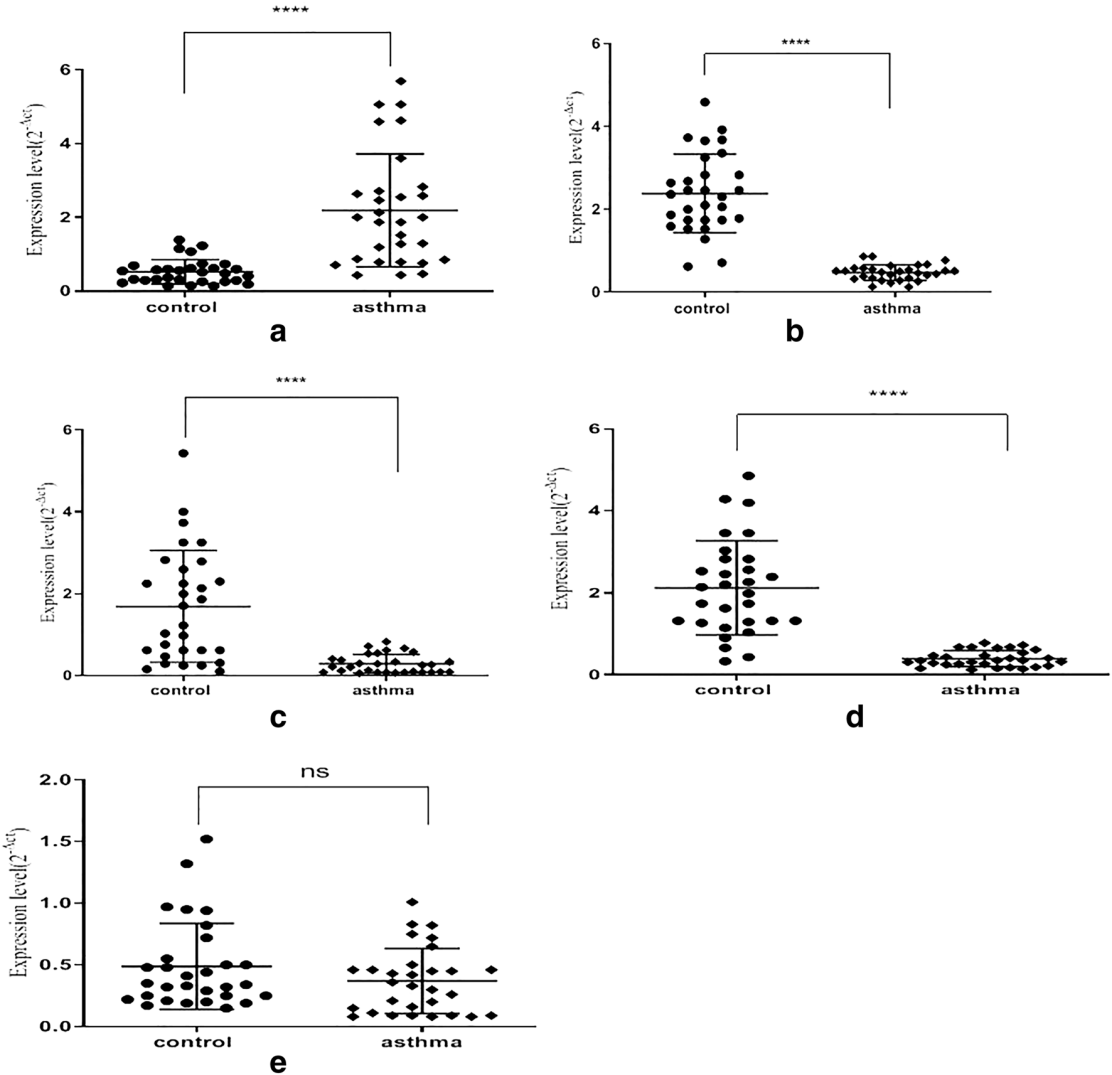
miRNAs expression level in plasma exosomes derived from severe asthmatic patients and healthy controls. **a** The qRT-PCR for miR-125b, **b** miR133b, **c** miR130a, **d** miR124 and **e** miR-125b-1-3p expression in severe asthma patients. The results are mean values of normal controls (n = 30) and asthma patients (n = 30). * indicates statistically significant difference between the groups

As shown in Fig. 2, the qRT-PCR data revealed that the expression of miR-124 in plasma exosomes was down-regulated in the patients suffering from severe asthma compared to that measured in normal individuals (normal controls; 2.12 ± 1.14 and patients, 0.39 ± 0.196). Likewise, the expression of miR-130a (normal controls, 1.69 ± 1.364; asthma patients: 0.29 ± 0.22), and miR-133b (normal controls: 2.38 ± 0.94, asthma patients: 0.47 ± 0.18) was significantly lower in patients compared to controls. In contrast, the expression of miR-125b in plasma exosomes of the asthma patients was substantially increased when compared to the samples obtained from normal controls (normal control, 0.52 ± 0.33; asthma patients, 2.188 ± 1.53). However, there was no significant difference in the expression of miR-125b-1-3p in patients and normal controls (normal: 0.48 ± 0.34; patients: 0.37 ± 0.26). The expression data presented above were normalized to internal control (U6) prior to analysis. The values are presented as Mean ± SD.

**Fig. 3:**
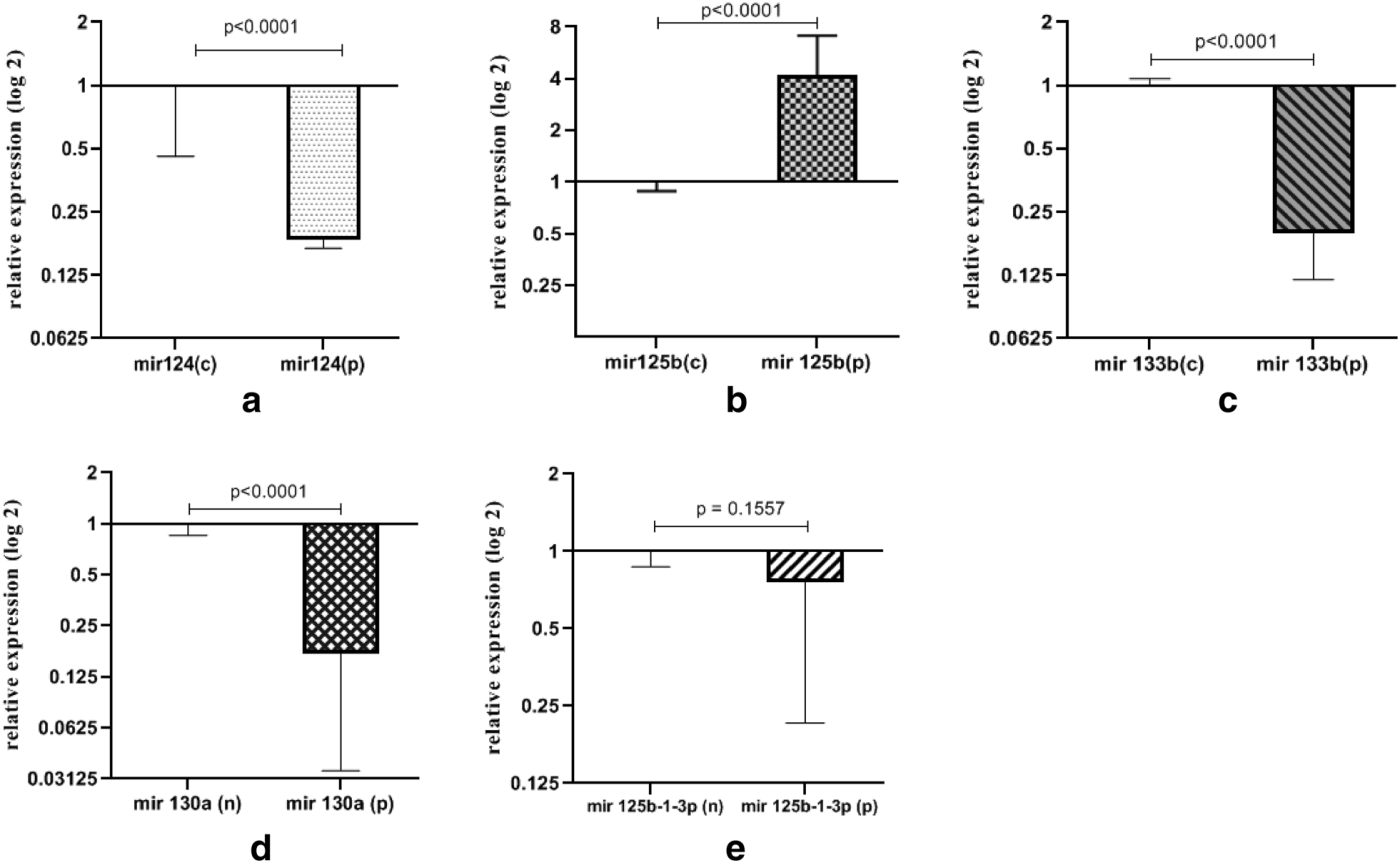
The relative expression (fold of change) of exosomal miRNAs in asthma patients. **a** miR-124, **c** miR-133b, and **d** miR-130a in severe asthmatic patients (n = 30) compared to control subjects (n = 30), are significantly down-regulated in patients (p < 0.0001). **b** miR-125b up-regulated in severe asthma and **e** miR-125b-1-3p had no significantly different compared to control. Data presented are mean ± SD. On the x-axis, (p) represents patients and (n) is for normal controls

**Fig. 4:**
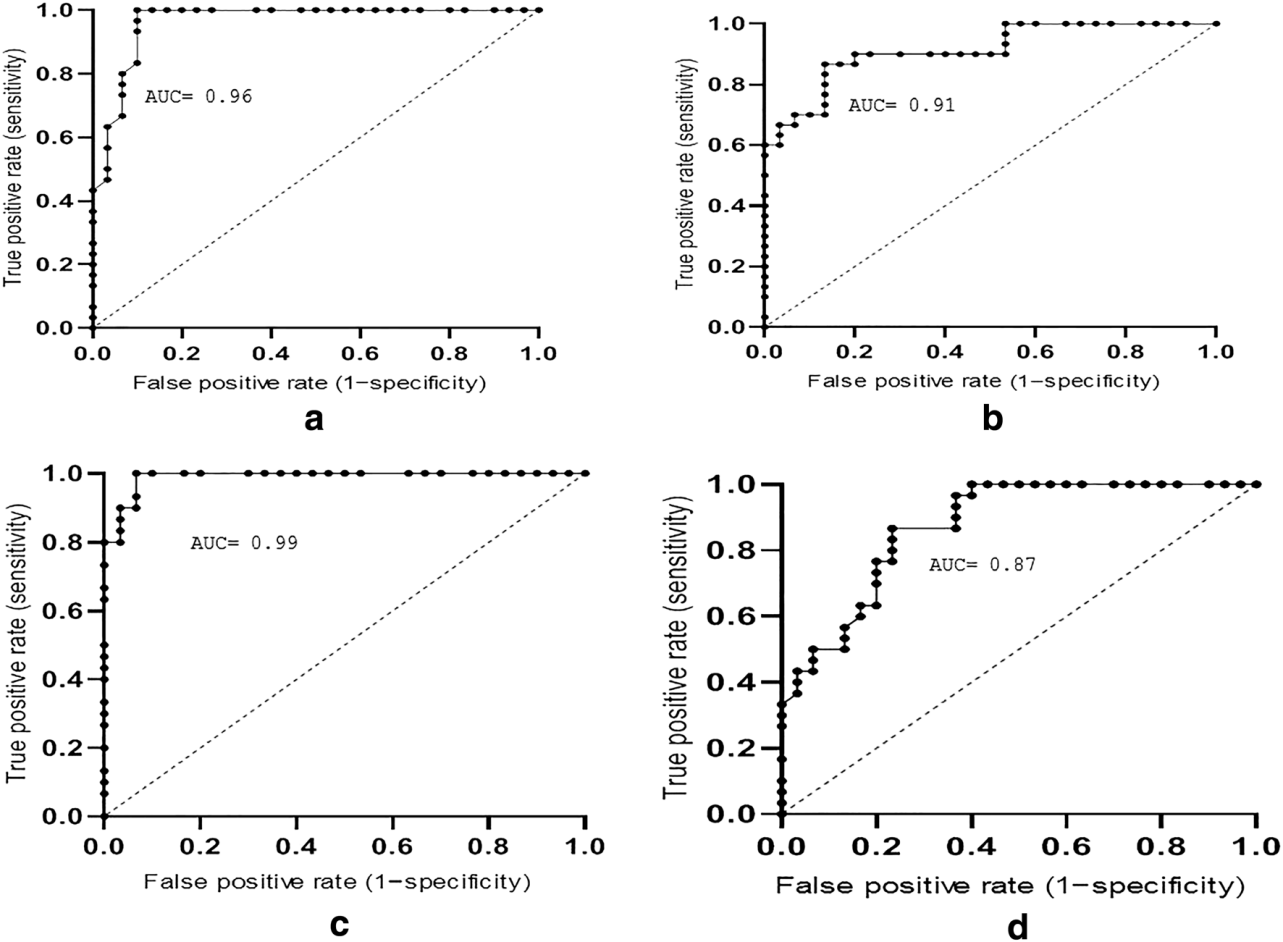
Diagnostic power of plasma exosomal miR-124 (**a**), miR-125b (**b**), miR-133b (**c**) and miR-130a (**d**) designated by ROC curve analysis. The results are shown as the area under ROC curve (AUC) for the sensitivity and specificity of each miRNA

The expression level of each microRNA is presented in terms of relative expression (fold change). The expression levels of miR-124, miR-133b, and miR-130a were significantly down-regulated in the patients' group compared to that of normal controls. Whereas, the expression of miR-125b was significantly up-regulated in the patients group when compared to the normal control group. The expression of miR-125b-1-3p remained unchanged in asthmatic patients compared to the healthy group (Table 2 and Fig. 3).The data obtained from the Receiver Operating Characteristic (ROC) curve show that expression of miR-124 with AUC = 0.96 (95% CI: 0.92–1.00), the miR-125b with AUC = 0.91 (95% CI: 0.84–0.98), the miR-133b with AUC = 0.99 (95% CI: 0.97–1.00) and miR-130a with AUC = 0.87 (95% CI: 0.79–0.96) were correlated with the asthma condition (p < 0.05) (Table 3 and Fig. 4).

**Table 2:** Relative expression (fold change) of different microRNAs in exosomes isolated from sera samples of asthma patients and controls

miRNA	Relative expression	P-value	overall expression
miR 124	0.19 ± 0.09	< 0.0001*	DOWN
miR 125b	4.17 ± 2.92	< 0.0001*	UP
miR 133b	0.20 ± 0.08	< 0.0001*	DOWN
miR 130a	0.17 ± 0.14	< 0.0001*	DOWN
miR 125b-3p	0.76 ± 0.54	0.156	No change

Purification and characterization of plasma exosomes have been described in the ‘Methods’ section. Fold change is presented as Mean ± SD. *p < 0.0001 is considered as significant. The overall expression shows differences in expression between the asthma patients and controls

**Table 3:** ROC curve predictive values obtained from AUC (Area under the curve) and CI (95% confidence interval) data in severe asthma samples

miRNA	AUC	CI 95% (p < 0.05)
miR 124	0.96	0.92–1.00
miR 125b	0.91	0.84–0.98
miR 133b	0.99	0.97–1.00
miR 130a	0.87	0.79–0.96

Furthermore, the expression level of exosomal miRNAs 124, 125b, 133b, and 130a was compared in terms of the type of medication given. In the patients given either inhaled corticosteroids (long-acting muscarinic antagonists (LAMAs) and long-acting beta-agonists (LABAs) or both inhaled corticosteroids and oral corticosteroids (1 mg prednisone per kg body weight) there was no significant difference in the expression in miR124, miR125b, miR133b, and miR130a (Results not shown).

### Comparison of serum levels of hs-CRP and total IgE in asthmatic patient and normal control groups

**Fig. 5:**
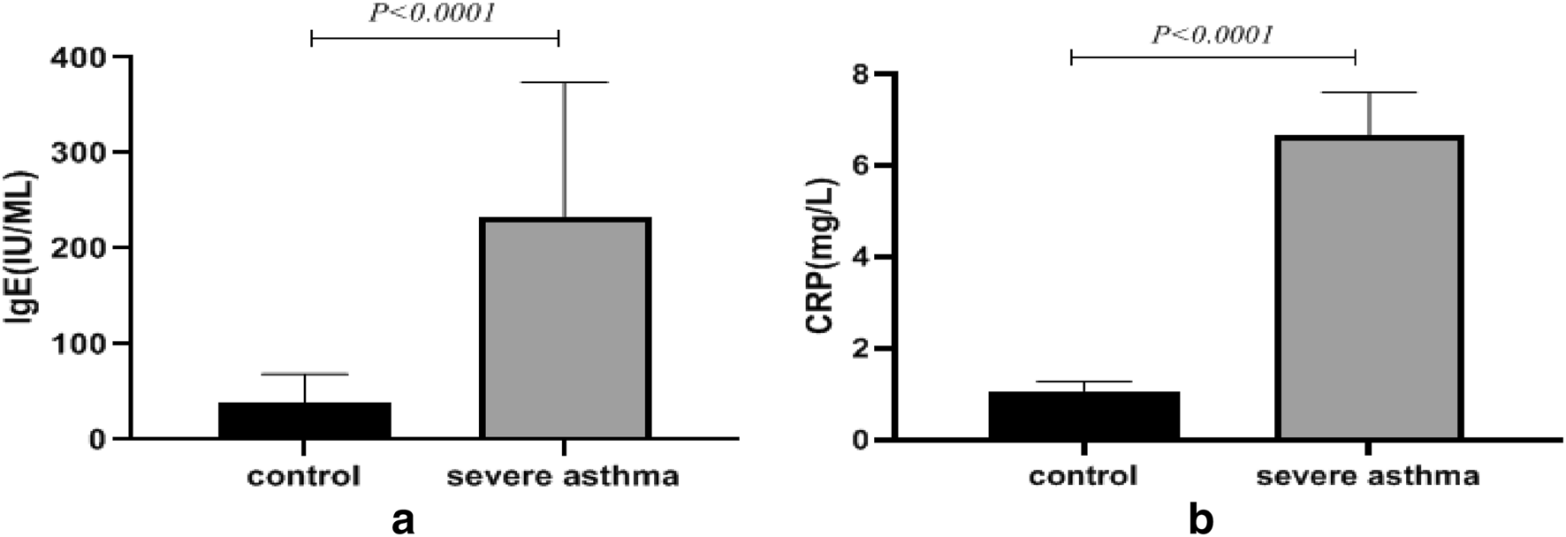
Comparison of serum levels of hs-CRP (section A) and total IgE (section B) in severe asthmatic patients and normal controls. The data are presented as mean values ± SD of all the samples in each group. The p-value < 0.05 is considered significant

Comparison of the serum levels of CRP and IgE between the normal controls and severe asthma patients are as shown in Fig. 5. Serum hs-CRP (controls = 1.075 ± 0.21; patients = 6.659 ± 0.94) and IgE (controls = 39.32 ± 5.33; patients = 23.8 ± 25.79) levels in asthma patients were significantly higher than that of healthy subjects (p < 0.0001).

### Correlation between the hs-CRP and IgE with the exosomal miRNAs

**Fig. 6:**
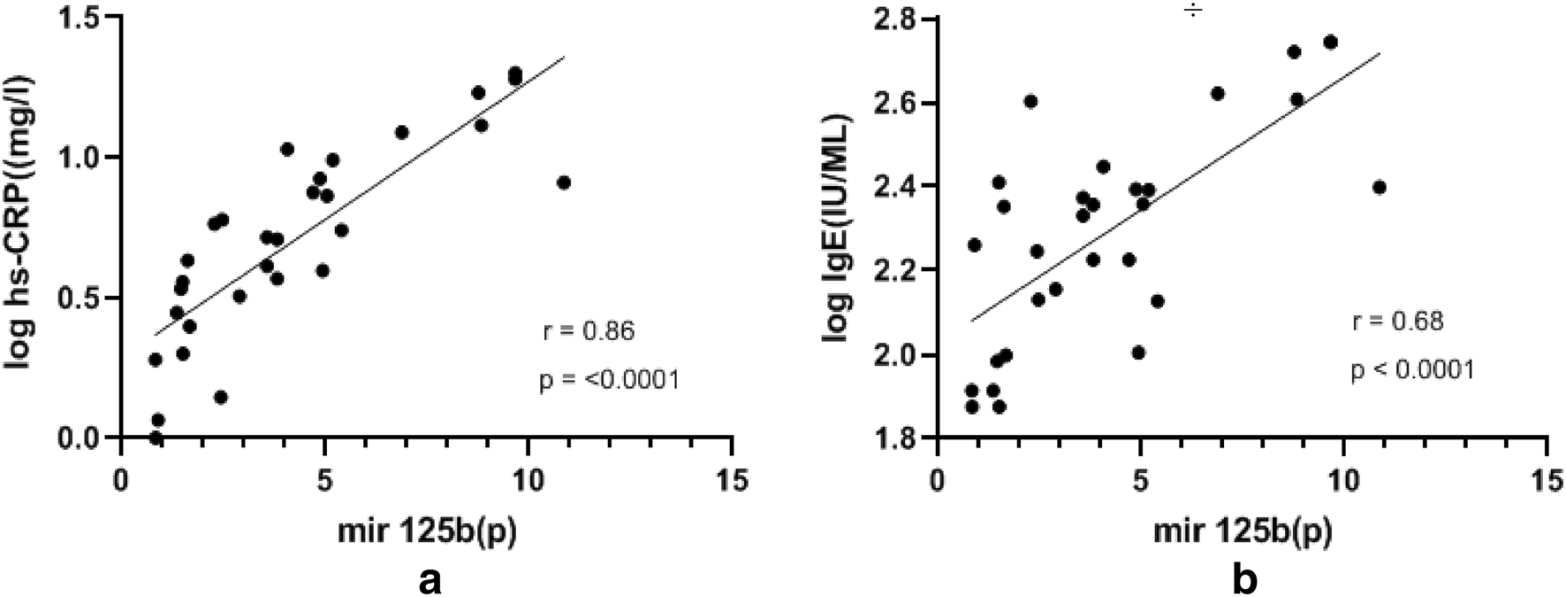
Relationship between the plasma exosomal miR 125b and inflammatory markers. Section **a** indicates the correlation of exosomal miR-125b and IgE and Section **b** shows the correlation of miR-125b and high sensitivity CRP. r = correlation coefficient, P-value was calculated by the Spearman’s rank correlation test. On the x-axis (p) represents patients

As shown in Table 4, correlation analysis showed that the expression level of exosomal miRNA125b is significantly correlated with the serum hs-CRP and total IgE levels (CRP; r = 0.86, p < 0.0001 and IgE, r = 0.68, p < 0.0001). However, the relationship between the expression of other miRNA molecules with the serum levels of IgE and hs-CRP in asthma patients was statistically not significant (Fig. 6).

**Table 4:** Relationship of the expression of exosomal miRNAs and serum hs-CRP and IgE in severe asthma patients

microRNAs	hs-CRP (log)		IgE level (log)	
	*r-value*	*P-value*	*r-value*	*P-value*
miR-124	0.22	0.23	0.07	0.73
miR-125b	0.86	< 0.0001	0.68	< 0.0001
miR-133b	0.29	0.12	0.35	0.06
miR-130a	0.34	0.06	0.29	0.12

p < 0.05 indicates the Spearman’s rank correlation with statistical significance

## Discussion

In the current study, serum levels of hs-CRP and total IgE as two mediators that are associated with inflammatory diseases with the expression of exosomal miRNAs (miR-124, miR-125b, miR-130a, miR-133b, and miR-125b-1-3p) were evaluated in severe asthma patients. The current results clearly show that among the miRNAs studied, the expression of miR-125b was significantly increased in plasma exosomal samples from asthma patients compared to that of normal control group. Interestingly, increased miR-125b expression was correlated with the inflammatory indices measured in serum. In fact the gene/pathway targets for these miRNAs are not precisely known, but an alteration in expression of a specific miRNA in severe asthma cases could be considered for further investigations. Up-regulation of the plasma exosomal miR-125b and its correlation with IgE and hs-CRP levels could be considered as an additional molecular marker for diagnosis of severe asthma. On the other hand, the possible interaction of miR-125b with inflammatory pathways could help our understanding of the mechanism(s) of the development of severe asthma. The interaction of miR-125b with inflammatory reaction in ulcerative colitis[47], encephalitis [48], and preeclampsia [31] further support our findings.

To our knowledge, this is the first report showing up-regulation of miR-125b in severe asthma patients and its link with serum levels of hs-CRP and total IgE, suggesting that miR-125b could be a good candidate as a molecular marker for the diagnosis of severe asthma besides clinical signs of disease.

Unlike miR-125b, the expression of serum exosomal miR-124, miR-130a, miR-133b was down-regulated in severe asthma patients when compared to samples collected from normal controls. Additionally, there was no significant relationship between the expression of these molecules with serum levels of IgE and CRP.

Therefore down-regulation of plasma exosomal miR-124, miR-130a and miR-133b in asthma patients may suggest that these molecules are probably negative regulators of inflammatory and allergic reactions and related target genes. These miRNAs were poorly related to hs-CRP and IgE levels in asthma patients.

Nevertheless association of the miR-125b expression with serum hs-CRP and total IgE in asthma patients may suggest the direct link between this particular miRNA with pathways and mediators involved in inflammatory reactions and allergy process. Studies showing up-regulation of miR-125b as the circulating microRNAs in patients with allergic rhinitis and asthma [37], further support our results in asthma patients have been reported.

In general, there are insufficient evidences to assign the expression of miRNAs to the certain disease conditions. However, reports show that sphingosine kinase 1 (SPHK1), which balances the inter-conversion of ceramide, sphingosine, and S1P can be directly targeted with miR-124 [49]. It appears that down-regulation of miR-124 in plasma exosomal samples from patients with severe asthma is linked to the regulation of the S1P pathway which is considered as a potent lipid mediator that can induce airway inflammation and asthma.

The relationship between the inflammatory factors and expression of miR-130a which regulates S1PR2 protein has also been described. Suppression of miR-130a is linked to inflammatory mediators such as TNF-α which may be responsible for increased inflammatory gene expression [30]. Therefore, it seems that a decrease in plasma exosomal miR-130a expression in asthma patients regardless of the high levels of serum IgE and CRP can be effective in increasing the inflammatory process in inflammatory diseases such as asthma. Decreased expression of circulating form of miR-133b in patients with allergic rhinitis and asthma [37], further support our data showing that miR-133b expression is decreased in plasma exosomes.

Involvement of the signal transducer and activator of transcription 3 (STAT3), which is activated by the S1PR1 pathway in causing inflammation and cancer has also been explained [38]. According to Cheng et al. miR-133b plays a significant role in controlling S1PR1 protein expression [29].

There are evidences which show that miRNA molecules are involved in inflammatory reactions. For example changes in miR-125b-1-3p which target S1PR1 are common in the pathogenesis of inflammatory diseases such as preeclampsia [28]. Also, it has been reported that down-regulation of miR-130a and miR-133b expression is not limited to the pathogenesis of severe asthma, but could be common for other inflammatory conditions.

The limited information obtained from this comparative study can be discussed in two directions. The impact of the alteration in expression of the miRNAs on inflammatory reactions in severe asthma conditions. Also the clinical implication of the miRNAs in the diagnosis of severe asthma. Our experience showed that selected miRNAs are differentially expressed in severe asthma cases and that the profile of expression of miRNAs differs in normal individuals and patients. A highly significant correlation between the expression of miR-125b and CRP/IgE levels (r = 0.86/r = 0.68; p < 0.0001) in asthma patients indicates the usefulness of this molecular marker for asthma diagnosis. Considerable up-regulation of miR-125b in the patients compared to normal controls is promising for discrimination of severe asthma from non-asthma individuals.

Based on the data obtained from the ROC curve, the changes in expression of miRNA-125b can be considered as a promising biomarker in diagnosis of severe asthma patients. However, the diagnostic value of a specific miRNA such as miRNA-125b relies on other factors such as the clinical stage and the clinical signs of the disease. The present study focused on the changes in miRNA expression in severe asthma patients compared to normal controls. However, the diagnostic application of miRNA expression can be confirmed by testing on more samples from patients suffering from mild, and moderate types of asthma. Moreover, further studies with other allergic and inflammatory conditions are needed to confirm the possible specificity of a miRNA to asthma conditions.

In conclusion, the data presented here may suggest that expression of miR-125b in the exosomal fraction of plasma and its correlation with serum CRP and total IgE is promising as a molecular marker for the diagnosis of severe asthma. Moreover, changes in the expression of microRNAs in plasma exosomes could be useful for discrimination of clinical phenotypes of asthma. It is to mention that obviously the development of a new marker for clinical applications including diagnosis and targeted therapy requires clinical validation and approval.

## Data Availability

Not applicable.

## Abbreviations

MiRNAs: MicroRNAs
S1P: Sphingosine-1-phosphate
GINA: Global Initiative for Asthma
ROC: Receiver operating characteristic
S1PR: Sphingosine-1-phosphate receptor
ASM: Airway smooth muscles
SphK1: Sphingosine kinase 1
SGPL1: Sphingosine-1-phosphate lyase 1
PBS: Phosphate-buffered saline
BSA: Bovine serum albumin
DLS: Dynamic light scattering
TEM: Transmission electron microscopy
PAP: Poly(A) polymerase
hs-CRP: High-sensitivity C-reactive Protein
IgE: Immunoglobulin E
BMI: Body mass index
BCA: Bicinchoninic acid
